# Associations between Oxidant/Antioxidant Status and Circulating Adipokines in Non-Obese Children with Prader–Willi Syndrome

**DOI:** 10.3390/antiox12040927

**Published:** 2023-04-13

**Authors:** Joanna Gajewska, Jadwiga Ambroszkiewicz, Katarzyna Szamotulska, Grażyna Rowicka, Małgorzata Strucińska, Witold Klemarczyk, Magdalena Chełchowska

**Affiliations:** 1 Department of Screening Tests and Metabolic Diagnostics, Institute of Mother and Child, Kasprzaka 17a, 01-211 Warsaw, Poland; 2 Department of Epidemiology and Biostatistics, Institute of Mother and Child, 01-211 Warsaw, Poland; 3 Department of Nutrition, Institute of Mother and Child, 01-211 Warsaw, Poland

**Keywords:** Prader–Willi syndrome, adipokines, oxidative stress, non-obese children, vitamins, fiber intake

## Abstract

Oxidative stress is implicated in the pathophysiology of Prader–Willi syndrome (PWS), but there are no data on these disorders in non-obese children with PWS. Therefore, the presented study examined total oxidant capacity (TOC), total antioxidant capacity (TAC), the oxidative stress index (OSI), and adipokine levels in 22 non-obese children with PWS during dietary intervention and growth hormone treatment compared with 25 non-obese healthy children. Serum concentrations of TOC, TAC, nesfatin-1, leptin, hepcidin, ferroportin, and ferritin were determined using immunoenzymatic methods. We found that TOC concentrations were higher by 50% (*p* = 0.006) in patients with PWS than in healthy children, but no significant differences in TAC concentrations were observed between these groups. The OSI was higher in children with PWS than in the controls (*p* = 0.002). We found positive associations between TOC values and the percentage of the Estimated Energy Requirement, body mass index (BMI) Z-score, percentage of fat mass, and leptin, nesfatin-1, and hepcidin concentrations in patients with PWS. A positive association was also found between the OSI and nesfatin-1 levels. These observations suggest that higher daily energy intake and weight gain may be accompanied by an increasing prooxidant state in these patients. Adipokines such as leptin, nesfatin-1, or hepcidin may also play a role in the prooxidant state in non-obese children with PWS.

## 1. Introduction

Prader–Willi syndrome (PWS) is a rare genetic condition caused by an absence of paternally active gene expression in the 15q11.2-13 region on the long arm of chromosome 15, either due to deletions from the paternal chromosome or maternal disomy [1]. Hypotonia and feeding difficulties in the first year of life are observed in children with PWS. Subsequently, these children develop an insatiable appetite leading to early childhood obesity [2]. It is suggested that aberrant adipocyte hypertrophy and an impaired adipogenesis process at an early phase is a potential pathological mechanism exacerbating hyperphagic obesity onset in patients with PWS [3]. Besides hyperphagia, other abnormalities are observed in patients with PWS due to hypothalamic dysfunction—such as short stature, hypersomnia, temperature instability, endocrine abnormalities including growth hormone and thyroid-stimulating hormone deficiencies, and hypogonadism. Developmental delay and behavioral problems also occur in Prader–Willi syndrome [4,5].

Data concerning oxidative stress in patients with PWS are rather scarce [6,7]. Ferretti et al. [6] demonstrated an increase in lipid hydroperoxide levels and a decrease in enzyme paraoxonase1 (PON1) activity in obese adults with PWS, in the absence of significant changes in plasma lipids. Other authors observed changes in the redox biomarker profile before antioxidant therapy in a non-obese child with PWS [7]. Management of Prader–Willi syndrome requires therapy with growth hormone (GH) and an extremely regulated diet to prevent obesity, avoid micro- and macronutrient deficiencies, and ensure optimal growth [8].

Adipose tissue is a metabolically active endocrine organ secreting many proteins, which may affect the metabolism of adipose tissue as well as other organs [9]. In many diseases—including obesity, diabetes, and atherosclerosis—increased adiposity is associated with low-grade inflammation, changes in the adipokine profile, and oxidative stress [10]. Imbalances in reactive oxygen species (ROS) homeostasis result in oxidative damage to biological molecules such as nucleic acids, lipids, and proteins [11]. ROS are also important mediators of inflammation and may promote the apoptosis process.

Excessive nutritional overload causes adipocyte hypertrophy and hyperplasia, which initiates oxidative stress and inflammatory reactions in adipose tissue [12]. Adipose tissue secretes various pro-inflammatory adipokines that modulate inflammation and insulin resistance. Leptin is an adipokine involved in the regulation of food intake, body mass, and energy expenditure, and plays a role in the pro-inflammatory immune response, angiogenesis, and lipolysis [13]. An increase in this adipokine is known to induce both nicotinamide adenine dinucleotide phosphate (NADPH) oxidase and inducible nitric oxide synthase causing the formation of reactive oxygen and nitric species, hence leptin may play an important role in the oxidative stress observed in children and adults with obesity [14].

Nesfatin-1—a multifunctional peptide (82 amino acids) with anorectic effects—is expressed in several regions of the hypothalamus and peripheral tissue, mainly in the stomach, pancreas, and adipose tissue [15]. Excess nesfatin-1 causes a loss of appetite, decrease in body weight, reduction of blood glucose levels, and stimulation of free fatty acid oxidation [16]. More recent evidence indicates that nesfatin-1 exerts antioxidant, anti-inflammatory, and antiapoptotic effects in different inflammation-related diseases [17]. The anti-inflammatory actions of nesfatin-1 were also demonstrated in several tissues, including human and murine models of acute lung inflammation [18,19]. However, some authors reported the pro-inflammatory properties of nesfatin-1 in human and murine chondrocytes and chronic obstructive pulmonary disease [20,21].

Hepcidin is a low-molecular-weight, antimicrobial peptide hormone regulating iron metabolism. This peptide is expressed mainly in the liver, but also in macrophages and adipose tissue under inflammatory conditions [22]. If iron is present in excess in cells and tissues, it disrupts redox homeostasis and catalyzes ROS production, leading to oxidative stress. Hepcidin regulates iron metabolism via the ferroportin (FPN) receptor, located mainly on the macrophages and duodenal enterocytes, triggering its degradation and hence regulating iron absorption [23].

Oxidative stress is involved in many disorders, including genetic diseases [24].

The intensity of oxidation processes can be assessed by total oxidant capacity (TOC), whereas enzymatic and non-enzymatic antioxidants can be evaluated by serum total antioxidant capacity (TAC). The oxidative stress index (OSI), which expresses the TOC/TAC ratio, is a valuable tool for assessing alterations in the oxidant/antioxidant balance. The estimation of the concentrations of each antioxidant separately as well as the total free radical scavenging capacity may also be useful in the assessment of the oxidant/antioxidant balance. Therefore, the presented study examined (a) TOC and TAC values and the oxidative stress index in non-obese children with PWS during the dietary intervention and growth hormone treatment in comparison with non-obese healthy children, (b) associations between the oxidant/antioxidant status and anthropometric and biochemical parameters including adipokines as well as dietary intake in children with PWS.

## 2. Materials and Methods

### 2.1. Patients

The study consisted of 22 Caucasian children with Prader–Willi syndrome aged 2–12 years, who were recruited between 2020 and 2022 from a group of consecutive patients seeking dietary counseling at the Institute of Mother and Child in Warsaw. The inclusion criteria of this study were: a genetically confirmed diagnosis of PWS, GH treatment for at least one year, GH therapy, and an energy-restricted diet at the time of inclusion. The exclusion criteria were: a body mass index (BMI) Z-score > 1 and a chronic secondary illness such as diabetes mellitus, liver, or kidney diseases. The control group included 25 healthy non-obese children (BMI Z-score <−1 + 1>) within the same age range as the group of patients with an adequate nutritional or dietary status according to Kułaga et al. [25] and Jarosz et al. [26]. The inclusion and exclusion criteria of this study, assessment of dietary intake as well as anthropometric measurements were described in detail in our previous study [27].

The three-day methodology (two weekdays and one weekend day) was used to assess the children’s dietary habits [28]. Nutritional analysis software (Dieta 5^®^, National Food and Nutrition Institute, Warsaw, Poland) was used to evaluate the average daily energy intake and the percentage of energy intake from carbohydrates, protein, and fat [29]. The age- and sex-specific percentage of Estimated Energy Requirement (EER) for total energy intake, Estimated Average Requirement (EAR) for vitamins A, B_12_, C, E, and iron, and Adequate Intake (AI) for fiber were calculated for each patient with Prader–Willi syndrome and each healthy child. The children in the present study only received standard supplementation with vitamin D.

The body mass index (BMI) of each child was converted to a BMI Z-score using Polish reference tables [25]. The body composition of all participants was measured by dual-energy X-ray absorptiometry (DXA) using Lunar Prodigy with pediatric software version 9.30.044 (General Electric Healthcare, Madison, WI, USA).

The presented study was performed in accordance with the Helsinki Declaration for Human Research. The protocol of this study was approved (No. 8/2020) by the Ethics Committee of the Institute of Mother and Child in Warsaw, Poland.

### 2.2. Biochemical Analyses

Venous blood samples were collected between 8:00 and 10:00 AM after an overnight fast. Blood in EDTA-containing tubes was analyzed immediately to determine red blood cell (RBC) count, hemoglobin (Hb), and mean corpuscular volume (MCV) using a hematology analyzer (Horiba ABX, Montpellier, France). Serum or EDTA plasma obtained after centrifuging the blood (2500× *g*, 4 °C, 10 min) was stored (−70 °C) until analysis was performed.

TOC and TAC values were measured in plasma using kits from Omnignostica Forschungs GmbH (Hoflein/Danube, Austria). The analytical sensitivity of TAC was 0.08 mmol/L, and the intra- and inter-assay CV were 5.0% and 6.9%, respectively. The sensitivity of TOC was 0.06 mmol/L, and the intra- and inter-assay coefficients of variation (CV) were 4.9% and 7.3%, respectively. The OSI index was expressed as the TOC/TAC ratio.

Serum FPN, hepcidin, leptin, and nesfatin-1 concentrations were determined by ELISA methods. Serum FPN was determined using the Human FPN ELISA kit (Elabscience, Houston, TX, USA) with anti-human FPN antibody, which had intra- and inter-assay CV of less than 5.4% and 6.1%, respectively. The concentration of nesfatin-1 was analyzed using the ELISA kit (Elabscience, Houston, TX, USA) with anti-human NES-1 antibody. The intra- and inter-assay CV for nesfatin-1 were 4.8% and 5.2%, respectively. Serum hepcidin concentration was measured using an ELISA kit (DRG, Marburg, Germany) with anti-human hepcidin-25. The intra- and inter-assay CV for hepcidin were less than 5.7% and 9.5%, respectively. The ELISA kit from DRG Diagnostics (Marburg, Germany) was used to analyze leptin concentration. The intra- and inter-assay CV for leptin were less than 9.6% and 9.1%, respectively. Serum ferritin, iron, and C-reactive protein (CRP) were measured using commercially available kits on a biochemical analyzer (ROCHE, Basel, Switzerland).

### 2.3. Statistical Analyses

The results are presented as means ± standard deviation (SD) for symmetrically distributed data or medians and interquartile range (25th–75th percentiles) for skewed distributed variables. The Kolmogorov–Smirnov test was used to evaluate distributions for normality. The non-parametric Mann–Whitney *U* test was used to evaluate the differences in anthropometric and biochemical parameters as well as dietary intake of patients with Prader–Willi syndrome and healthy children without obesity.

Logistic regression was used to study the differences between the groups after adjustment. For correlation analysis, Spearman ρ was applied. Quantile regression was used to fit regression lines. A *p*-value of <0.05 was considered to be statistically significant. Statistical analysis was performed using IBM SPSS v.25.0 software (SPSS Inc., Chicago, IL, USA).

## 3. Results

Table 1 shows similar values of height, weight, BMI, BMI Z-score, and percentage of fat mass in children with PWS and healthy non-obese children.

Serum TOC concentrations were higher by about 50% (*p* = 0.006) in patients with PWS than in healthy children, but no significant differences in serum TAC concentrations were observed between these groups. OSI was higher in children with PWS than in the controls (*p* = 0.002). Among the analyzed adipokines, higher concentrations of nesfatin-1 (*p* = 0.003) were observed in patients than in healthy children. No differences in leptin, hepcidin, ferroportin, ferroportin/hepcidin, and ferritin/hepcidin ratios were found between both groups. Ferritin concentrations were higher by about 50% (*p* = 0.001) in patients with PWS than in the controls but serum iron concentrations were unchanged. In children with PWS, the hematological parameters—such as hemoglobin, RBC, and MCV—were within the reference range and similar to the normal-weight group. The median values of the marker CRP were also similar in both studied groups, being within the normal range (below 5 mg/L).

The daily energy intake and the percentage of EER were significantly lower (*p* = 0.001) in children with PWS compared with controls (Table 2).

Recommended daily energy and nutrients intakes (1–3/4–6/7–9/10–12 girls/10–12 boys, years) according to Jarosz [24]: energy (/1000/1400/1800/2100/2350 kcal/day), protein (0–2 years, 5–15%; 3–18 years, 10–20%), carbohydrate (1–18 years, 45–65%), fat (1–3 years 35–40%; 4–18 years, 20–35%), iron (3/4/4/7/7 mg/day), vitamin A (280/300/350/430/450 µg/day), vitamin B_12_ (0.7/1.0/1.5/1.5/1.5 µg/day), vitamin C (30/40/40/40/40 mg/day), vitamin E (6/6/7/8/10 mg/day), fiber (10/14/16/19/19 g/day).

The percentage of energy from proteins was significantly higher (*p* < 0.001) in patients with PWS than in healthy children. The proportions of fat and carbohydrates in the daily energy intake were similar in both groups. The differences between both studied groups concerning vitamin A intake and the percentage of the EAR for this vitamin were not statistically significant. The diet of children with PWS and the controls contained similar amounts of vitamin E and the mean value of EAR percentage for this vitamin in both groups was consistent with the recommendations concerning daily intake [20]. The diet of children with PWS contained a higher intake of iron (*p* = 0.017) and vitamins B_12_ (*p* = 0.033) and C (*p* < 0.001) than that of healthy children. The percentage of EAR for iron, vitamin B_12_, and vitamin C intake was also significantly higher in patients than in controls (*p* < 0.013; *p* = 0.006; *p* < 0.001, respectively). Similar values of fiber intake were observed in both studied groups.

There are statistically nonsignificant correlations between TOC (rho = 0.033, *p* = 0.832), TAC (rho = 0.351, *p* = 0.111), and OSI (rho = −0.247, *p* = 0.276) and age in the whole group with PWS. However, there is a statistically significant correlation between nesfatin-1 (rho = 0.548, *p* = 0.008) and age in the group with PWS. There are statistically nonsignificant correlations between TOC (rho = −0.206, *p* = 0.372), TAC (rho = 0.296, *p* = 0.215), OSI (rho = −0.323, *p* = 0.145), and nesfatin-1 (rho = 0.215, *p* = 0.329) and age in the whole control group.

In children with PWS, we observed positive associations between TOC and leptin (*p* = 0.022), nesfatin-1 (*p* = 0.021), and hepcidin (*p* = 0.012) (Table 3). Additionally, we found a positive association between OSI and nesfatin-1 in these patients.

Negative associations in children with PWS were observed between TOC and the ferritin/hepcidin ratio (*p* = 0.009). Any associations with TOC, TAC, and OSI were found for ferroportin, the ferroportin/hepcidin ratio, and ferritin in these patients.

**Figure 1:**
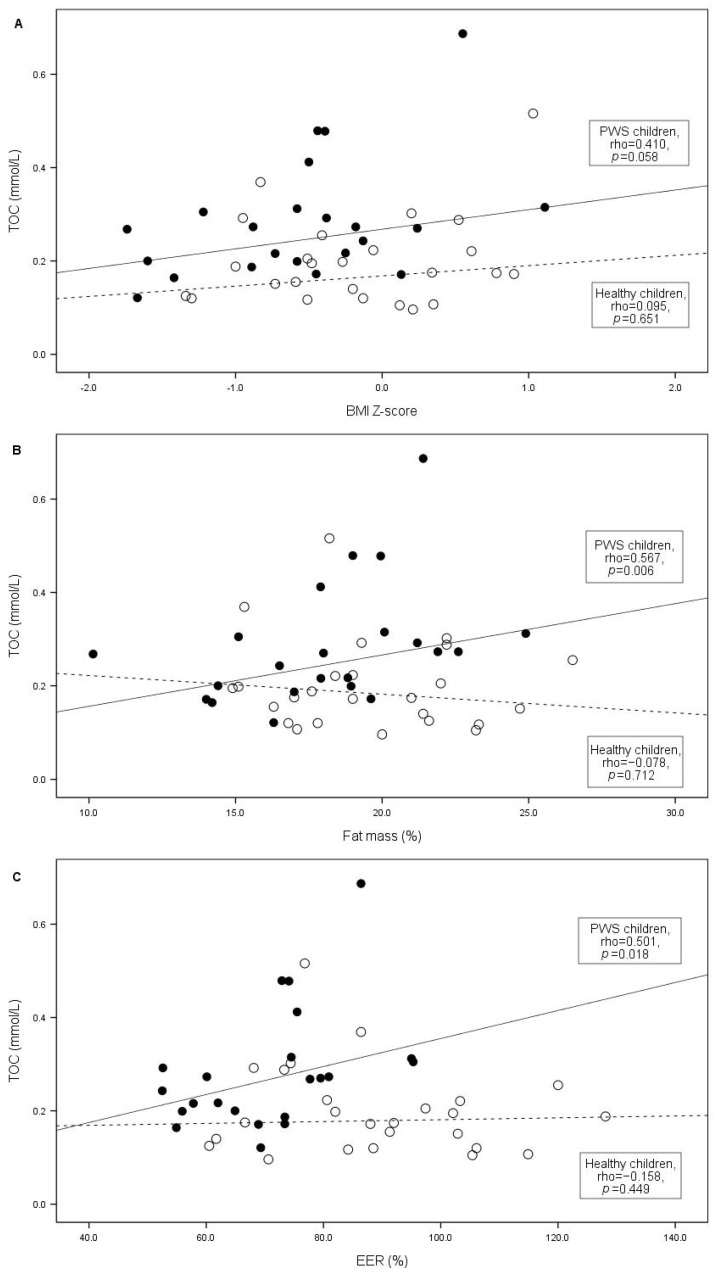
TOC values and BMI Z-score (**A**), fat mass percentage (**B**), and EER percentage (**C**) in children with PWS and controls. Black circles—PWS children, white circles—healthy children. Lines represent quantile regression.

In patients with PWS, we obtained positive associations between TOC and BMI Z-score (borderline value; ρ = 0.410, *p* = 0.058) (Figure 1A). However, after adjusting for BMI Z-score, TOC values were significantly higher in patients with PWS than in the controls (*p* = 0.011).

Positive associations were also found between TOC values and percentage of fat mass (ρ = 0.567; *p* = 0.006) and percentage of EER (ρ = 0.501; *p* = 0.018) in children with PWS (Figure 1B,C). These associations were not observed in the control group.

**Figure 2:**
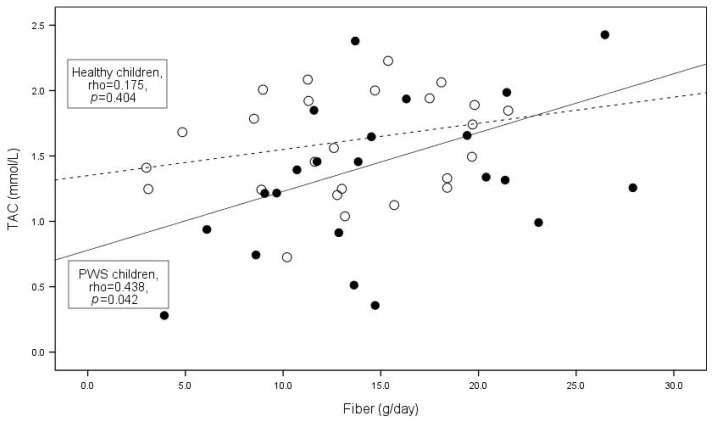
Associations between TAC values and fiber intake in children with PWS and controls. Black circles—PWS children, white circles—healthy children. Lines represent quantile regression.

We also found positive associations between TAC values and fiber intake (ρ = 0.438; *p* = 0.042) in patients with PWS (Figure 2).

When analyzing the relations between EER percentage and fiber intake, we found positive associations between these parameters (ρ = 0.438; *p* = 0.042) in patients with PWS.

## 4. Discussion

In the presented study, we found a disturbed balance between prooxidants and antioxidants in children with PWS, regardless of age, despite strict dietary supervision, therapy with growth hormone, and maintaining normal body weight. The prooxidant state observed in these patients results from increased oxidative potential (TOC) rather than from a deficit of antioxidant potential (TAC). According to the literature data, oxidative stress may be implicated in the pathophysiology of PWS [30]. The changes in the structural, enzymatic, and transcriptional mitochondrial components found in an imprinting center deletion mouse model of PWS play a role in mitochondrial dysfunctions, which may lead to disturbances in energy metabolism, ROS production, and apoptosis processes in this genetic disease. Ferretti et al. [6] found higher levels of lipid hydroperoxide, lower activity of PON1, alterations in HDL composition and functional properties as well as higher levels of CRP and pro-inflammatory cytokines in 15 obese adults with PWS. Oxidative stress was also observed in a non-obese girl with PWS during GH therapy using a redox biomarker profile including total hydroperoxides, non-protein-bound iron, thiols, advanced oxidation protein products, and isoprostanes [7]. Therapy with the antioxidant agent potassium ascorbate with ribose (PAR) decreased oxidative stress biomarker levels until their normalization in this patient.

The obesity manifested in patients with PWS may be associated with an inflammatory condition characterized by a rise in pro-inflammatory cytokines, which contribute to increased oxidative stress [31,32]. These authors observed higher levels of C-reactive protein and interleukin-6 and 18 in obese adults with PWS. Although the children with PWS in our study were not obese, we found positive associations between BMI Z-score, percentage of fat mass, and TOC values in these patients. Moreover, an association was also observed between TOC values and EER percentage. These observations suggest that higher daily energy intake and weight gain may be accompanied by an increasing prooxidant state in these patients.

Oxidative stress conditions in obese subjects are associated with raised serum concentrations of pro-inflammatory adipokines, such as leptin [33]. Many authors observed hyperleptinemia in obese children with PWS [34,35], but we found low values of leptin in our non-obese children with PWS and similar to the normal-weight healthy children. We also observed positive associations between leptin levels and TOC values in children with PWS. It seems that this adipokine may be associated with the prooxidant state found in the studied group with PWS.

Among the studied adipokines, we found higher nesfatin-1 levels and their positive associations with TOC and OSI in children with PWS. Literature data on the role of nesfatin-1 in the redox balance are contradictory. Gharanei et al. [36] suggested the anti-inflammatory effects of nesfatin-1 in murine subcutaneous adipose tissue showing that nesfatin-1 may activate the nuclear factor erythroid 2–related factor 2 (NRF2) pathway and subsequently inhibit NF-κB pro-inflammatory activity. Moreover, this adipokine can reduce the expression of nitric oxide (NO) and prostaglandin E2 (PGE2) by suppressing inducible nitric oxide synthase (iNOS) and cyclooxygenase (COX)-2, protecting the gastric mucosa [37]. In addition, nesfatin-1 exerts its antioxidant effect by decreasing myeloperoxidase and malondialdehyde levels and increasing antioxidants such as superoxide dismutase, catalase, and glutathione in renal ischemia-reperfusion injury in rats [38]. However, according to Leivo-Korpela et al. [21], plasma concentrations of nesfatin-1 correlated with circulating levels of interleukin 6 (IL-6) and tumor necrosis factor-𝛼 (TNF-𝛼) and interleukin 8 (IL-8), suggesting that these adipokines may play a role in the systemic inflammation in chronic obstructive pulmonary disease (COPD). Other authors observed that TNF-𝛼, IL-6, and insulin increased nesfatin-1 secretion in human and murine adipose tissue [39]. Our study also shows the associations between nesfatin-1 and the prooxidant state in non-obese children with PWS, but a compensatory role of this adipokine in promoting the redox balance cannot be ruled out.

There are significant relationships between hepcidin, iron, inflammation, and oxidative stress [22,40]. Higher concentrations of hepcidin were found in non-PWS obese children and adolescents by many authors [41,42,43]. Although, Chang et al. [44] observed lower hepcidin values in these patients compared with the control group. Some authors found that higher hepcidin levels were associated with lower or unchanged iron and ferritin values in obese children [41,45]. In our study, we found significantly higher iron and vitamin B_12_ intakes and higher concentrations of ferritin, but similar concentrations of hepcidin, ferroportin, and serum iron in non-obese children with PWS compared with healthy subjects. Hepcidin was positively associated but the ferritin/hepcidin ratio was negatively associated with TOC values, which may result in increased iron content in the macrophages, liver, and adipose tissue in children with PWS [46]. This may produce the conditions for the adverse effect of iron overload such as oxidative stress in patients with higher values of hepcidin.

Suboptimal dietary intake of several essential nutrients—such as iron, zinc, tocopherol, and fiber—was described in patients with PWS by many authors [47,48,49]. It is suggested that this may result mainly from the reduced energy intake in these patients [49]. However, according to Mackenzie et al. [48], adolescents with PWS had similar nutrient intakes to adolescents without PWS despite a lower energy intake. It could be attributed to the higher diet quality, but some young people with PWS were nevertheless at risk of inadequate fiber, vitamin, and mineral intake. Many studies showed that children with PWS require a 20–40% reduction in energy intake to maintain a healthy body weight [50]. In our study, children with PWS required about 30% less energy to maintain a healthy weight. Despite a low-energy diet, these patients consumed adequate amounts of protein and proportions of carbohydrates and fat. Moreover, our children with PWS consumed similar amounts of vitamins A and E and higher amounts of vitamins B_12_ and C in comparison with healthy non-obese children. It is known that fat-soluble vitamins and vitamin C act as free radical scavengers, which belong to the most important components of the antioxidant cell defense system as non-enzymatic antioxidants [51]. In addition, we found positive associations between EER% and fiber intake in these patients. Fiber may play a role in the regulation of inflammation in PWS children [52]. According to Li et al. [52], a gut microbiota-targeted dietary intervention with a high-fiber diet may improve the immune status of children with PWS and children with simple obesity. Zang et al. [53] observed that a high-fiber diet could reduce body weight, serum antigen load, and inflammation levels in these patients. However, despite the control-like antioxidant potential and adequate supply of antioxidant vitamins and fiber, the patients with PWS in our study showed a disturbed balance between prooxidants and antioxidants. It seems that the need for additional supplementation of certain nutrients can be considered in these patients.

The possibility of using antioxidant supplementation in oxidative stress-related diseases has been intensively studied for many years [54]. The results of these studies are still ambiguous, as moderate consumption of antioxidants may be beneficial, while excessive consumption of antioxidants may have adverse effects on health. Therefore, it is advisable to monitor the consumption of antioxidants and their blood levels in children with PWS during supplementation.

The present study had several limitations. First, we had a relatively small number of participants owing to the rarity of Prader–Willi syndrome. However, the study group was homogeneous in terms of therapy involving GH treatment and a low-energy diet as well as anthropometrically and biochemically. The second limitation of this study was its cross-sectional nature and the absence of a prospective longitudinal analysis, which is needed to examine the relationship between the redox balance and clinical outcomes in children with PWS. However, this is the first study to investigate the oxidant/antioxidant status of non-obese children with PWS during GH treatment and dietary intervention. Finally, enzymatic (glutathione-linked enzymes) and other non-enzymatic antioxidants (vitamins, glutathione) might also be useful in evaluating the total antioxidant capacity in patients with PWS. We will consider these issues in our next studies on patients with PWS.

## 5. Conclusions

In conclusion, we observed a disturbed balance between prooxidants and antioxidants in non-obese children with PWS during therapy with GH and a low-energy diet. The higher OSI observed in these patients results from an increased total oxidant capacity rather than from a deficit of total antioxidant capacity. We found positive associations between TOC values and leptin, nesfatin-1, hepcidin, percentage of Estimated Energy Requirement, BMI Z-score, and percentage of fat mass in patients with PWS. Moreover, a positive association was also observed between OSI and nesfatin-1 levels. These observations suggest that higher daily energy intake and weight gain may be accompanied by an increasing prooxidant state in these patients. In addition, adipokines such as leptin, nesfatin-1, or hepcidin may also play a role in the prooxidant state in non-obese children with PWS.

## Figures and Tables

**Table 1:** Anthropometric and biochemical characteristics of children with PWS and healthy children.

	Children with PWS n = 22	Healthy Children n = 25	*p*-Value
Age (years)	5.7 ± 3.3	6.3 ± 3.2	0.622
Male (%)	50.0	48.0	1.000
Anthropometric parameters			
Height (cm)	110.4 ± 24.2	116.7 ± 21.8	0.400
Weight (kg)	18.3 (11.3–24.8)	18.0 (15.8–30.1)	0.292
BMI (kg/m^2^)	15.2 ± 1.4	16.0 ± 1.7	0.093
BMI Z-score	−0.55 ± 0.72	−0.17 ± 0.67	0.107
Fat mass (%)	18.2 ± 3.4	19.6 ± 3.1	0.197
Biochemical measurements			
TOC (mmol/L)	0.269 (0.196–0.313)	0.175 (0.123–0.239)	0.006
TAC (mmol/L)	1.303 ± 0.584	1.581 ± 0.392	0.107
OSI	0.180 (0.138–0.346)	0.108 (0.082–0.190)	0.002
Leptin (ng/mL)	2.64 (1.12–3.23)	1.61 (0.88–2.23)	0.257
Nesfatin-1 (ng/mL)	1.55 (0.46–1.91)	0.53 (0.31–0.85)	0.003
Hepcidin (ng/mL)	5.71 (4.85–7.60)	5.70 (3.29–9.95)	0.533
Ferroportin (ng/mL)	17.0 (12.1–21.1)	14.4 (10.8–21.0)	0.586
Ferritin (ng/mL)	35.5 (28.0–54.3)	24.0 (18.6–37.5)	0.001
Ferroportin/Hepcidin	2.59 (1.53–3.60)	2.83 (1.91–4.20)	0.533
Ferritin/Hepcidin	6.89 (3.90–9.37)	4.84 (2.89–9.51)	0.365
Iron (µmol/L)	16.2 ± 4.3	16.3 ± 6.4	0.536
RBC (×10^6^/µL)	4.58 ± 0.25	4.72 ± 0.23	0.054
Hb (g/dL)	13.08 ± 0.84	13.03 ± 0.73	0.612
MCV (f/L)	83.8 ± 4.7	81.5 ± 3.5	0.143
CRP (mg/L)	0.55 (0.10–0.88)	0.35 (0.19–0.60)	0.706

Results are presented as means ± standard deviations for normally distributed data, or medians and interquartile ranges (25th–75th percentiles) for non-normally distributed variables. BMI = body mass index; RBC = red blood cells; Hb = hemoglobin; MCV = mean corpuscular volume; CRP = C-reactive protein; TOC = total oxidant capacity; TAC = total antioxidant capacity; OSI = oxidative stress index (TOC/TAC).

**Table 2:** Daily energy and nutrient intake of the examined children with PWS and healthy children.

Parameter	Children with PWS n = 22	Healthy Children n = 25	*p*-Value
Energy (kcal/24 h)	999 ± 343	1366 ± 371	0.001
Energy (% of EER)	70.8 ± 12.5	89.0 ± 18.2	0.001
Proteins (% of energy intake)	16.9 ± 4.3	13.1 ± 1.8	<0.001
Carbohydrates (% of energy intake)	51.9 ± 7.0	54.0 ± 6.1	0.237
Fat (% of energy intake)	29.7 ± 5.1	32.1 ± 5.3	0.215
Iron (mg/day)	9.86 ± 3.97	7.51 ± 1.86	0.017
Iron (% of EAR)	240.3 ± 91.8	175.6 ± 54.8	0.013
Vitamin A (µg/day)	976.9 (590.8–1207.5)	531.0 (307.8–1700.8)	0.162
Vitamin A (% of EAR)	291.9 (204.7–371.7)	171.1 (102.6–477.2)	0.086
Vitamin B_12_ (µg/day)	3.24 ± 1.57	2.31 ± 0.81	0.033
Vitamin B_12_ (% of EAR)	318.3 ± 129.0	207.7 ± 80.7	0.006
Vitamin C (mg/day)	110.1 ± 49.0	57.2 ± 25.4	<0.001
Vitamin C (% of EAR)	319.5 ± 147.9	148.5 ± 69.3	<0.001
Vitamin E (mg/day)	7.66 ± 3.04	6.95 ± 3.46	0.347
Vitamin E (% of EAR)	117.9 ± 50.8	101.9 ± 49.5	0.225
Fiber (g/day)	15.0 ± 6.4	13.3 ± 5.2	0.403
Fiber (% of AI)	111.2 ± 37.5	92.1 ± 33.8	0.099

The results are presented as means ± standard deviations for normally distributed data or medians and interquartile ranges (25th–75th percentiles) for non-normally distributed variables. % of EER = percentage of Estimated Energy Requirement; EAR = Estimated Average Requirement; AI = adequate intake (AI) for fiber The data are presented as recommended daily energy and nutrient intake according to Jarosz et al. [26].

**Table 3:** Associations between total oxidant and antioxidant capacity, OSI and serum adipokines, and acute phase protein concentrations in patients with PWS.

Parameter	TOC		TAC		OSI	
	ρ_Spearman_	*p*-Value	ρ_Spearman_	*p*-Value	ρ_Spearman_	*p*-Value
Leptin	0.485	0.022	−0.203	0.366	0.394	0.070
Nesfatin-1	0.487	0.021	−0.224	0.316	0.445	0.038
Hepcidin	0.525	0.012	0.136	0.546	0.168	0.454
Ferroportin	−0.058	0.799	−0.169	0.452	0.132	0.557
Ferritin	0.016	0.945	0.210	0.347	−0.108	0.632
Ferroportin/Hepcidin	−0.389	0.073	−0.159	0.481	−0.068	0.764
Ferritin/Hepcidin	−0.544	0.009	−0.025	0.911	−0.282	0.203

TOC = total oxidant capacity; TAC = total antioxidant capacity; OSI = oxidative stress index (TOC/TAC).

## Data Availability

The data presented in this study are available upon reasonable request to the corresponding author. The data are not publicly available due to ethical reasons.
